# Endothelin 1 gene is not a major modifier of chronic kidney disease advancement among the autosomal dominant polycystic kidney disease patients

**Published:** 2015-12-09

**Authors:** Shiva Nagendra Reddy Annapareddy, Ramprasad Elumalai, Bhaskar V.K.S. Lakkakula, Gnanasambandan Ramanathan, Soundararajan Periyasamy

**Affiliations:** ^1^Department of Nephrology, Sri Ramachandra University, Chennai, India; ^2^Department of Biomedical Sciences, Sri Ramachandra University, Chennai, India; ^3^Sickle Cell Institute Chhattisgarh, Raipur, India

**Keywords:** Hypertension, Chronic kidney disease, Autosomal dominant polycystic kidney disease

## Abstract

**Introduction:** Autosomal dominant polycystic kidney disease (ADPKD) is characterized by the presence of numerous cysts in the kidney and manifest with various renal and extra-renal complications leading to ESRD. Endothelin may contribute to various renal and extra-renal manifestations pointing to genetic and environmental modifying factors that alter the risk of developing chronic kidney disease (CKD) in ADPKD. In the present study we investigated six genes coding for endothelin 1 ( *EDN1 *) tagging-single nucleotide polymorphisms (tag-SNPs) to unravel the *EDN1* gene modifier effect for renal disease progression in ADPKD.

**Materials and Methods:** The tag-SNPs were genotyped using FRET-based KASPar method in 108 ADPKD patients and 119 healthy subjects. Cochran-Armitage trend test was used to determine the association between ADPKD and *EDN1* tag-SNPs. Multivariate logistic regression analysis was performed to assess the effect of tag-SNPs on CKD progression. The relationship between different CKD stages and hypertension and their interaction Mantel-Haenszel stratified analysis was performed.

**Results:** All loci are polymorphic and followed Hardy-Weinberg equilibrium. Distribution of *EDN1* genotypes and haplotypes in control and ADPKD is not statistically significant. Five SNPs covering 3.4 kb forming single LD block, but the LD was not strong between SNPs. The *EDN1* genotypes are not contributing to the CKD advancement among the ADPKD patients.

**Conclusion:** These results suggest that the *EDN1* gene is not a major modifier of CKD advancement among ADPKD patients.

**Implication for health policy/practice/research/medical education:**

The present study evaluated gene coding for endothelin 1 (EDN1) gene polymorphism in autosomal dominant polycystic kidney disease (ADPKD) with chronic kidney disease (CKD) patients and healthy subjects. Our study demonstrated that the distribution of EDN1 genotypes in control and ADPKD is not statistically different. These results imply that the EDN1 gene is not a major modifier of CKD advancement in ADPKD.

## Introduction

Autosomal dominant polycystic kidney disease (ADPKD) is one of the most common inherited diseases of the kidney (1). ADPKD is characterized by the formation of fluid filled cysts in both kidneys eventually replacing almost all normal renal parenchyma leading to a decline in renal function. Although ADPKD is a systemic disease it exhibit several extra-renal manifestation such as hypertension, hepatic cysts, pancreatic cysts and cerebral aneurysms during the course of their disease (2). Hypertension occurs prior to loss of renal function in about 70% of patients with ADPKD (3). ADPKD patients are 50% more likely to reach end-stage renal disease (ESRD), which accounts for 10% of the ESRD cases (4). Hence ADPKD patients require dialysis or renal transplantation at the age of 55 years (5). Although the inﬂuence of environmental factors on chronic kidney disease (CKD) progression in ADPKD has not been clariﬁed, the intra-familial variability in the age at ESRD suggests a potential role for modifier genes influencing renal disease progression in ADPKD.

Hypertension is one of the most common early manifestations of ADPKD and correlated with the progressive kidney enlargement. The renin–angiotensin–aldosterone system (RAAS) and the endothelin (ET) system entail the most potent vasopressor mechanisms identified to date. It has been suggested that the endothelin-1 (ET-1) is one of the major disease-inducing factors in renal disease. ET-1 causes renal vasoconstriction with a concomitant decrease in renal blood flow and glomerular filtration rate (6). ET-1 stimulates glomerular cell proliferation, extracellular matrix deposition and alter the expression of several genes (7). Of the three endothelin peptides, endothelin-1, -2, and -3, endothelin-1 (*EDN1*) is the major renal isoform produced by and acting on the mesangial cells (8). The gene coding for endothelin 1 (*EDN1*) has been localized to chromosome 6p24-p23 (9). ET-1 mRNA encodes a 212-amino acid pre-propeptide that is cleaved to yield 38-amino acid big ET-1. The mature 21-amino acid ET-1 is generated by a specific enzymatic cleavage of the big-EDN1 at Trp-21-Val-22 (10).

Several genetic variants of *EDN1*, which may influence the hereditary risk of cardiovascular diseases such as coronary heart disease, hypertension, and ventricular arrhythmia have already been identified (11-13). In view of the role of hypertension on the progression of CKD in ADPKD, the gene polymorphisms of *EDN1* are of great interest.

## Objectives

In the present study we investigated the EDN1 tagging-single nucleotide polymorphisms (tag-SNPs) to unravel the EDN1 gene modifier effect for renal disease progression in ADPKD.

## Materials and methods

### Subjects

This study consisted of 108 ADPKD patients confirmed through well-established ultrasound-based criteria (14) and 119 controls without any kidney related diseases. The samples used in this study were collected from Department of Nephrology, Sri Ramachandra University, Chennai, India. The demographic, clinical, and biochemical variables were obtained from all the participants. Modification of diet in renal disease *(*MDRD*)* formula was used to determine the glomerular filtration rate (15) and total number of cysts were identified by ultrasound examination. The CKD stage in all the ADPKD patients was determined according to the national kidney foundation recommendations (16) and patients were divided into two groups such as early stages* (*CKD 1-3 stages) and advanced (CKD 4 & 5 stage) stages (17). Genomic DNA from the samples was extracted by phenol chloroform extraction and ethanol precipitation protocol (18).

### SNP selection and genotyping

The *EDN1* tag-SNPs were selected using SNPinfo a web-based tool (http://www.niehs.nih.gov/snpinfo) (19). The tag-SNPs selected are localized in the *EDN1* gene including 1000 base pair (bp) 5’- and 3’ flanking regions with a minor allele frequencies (MAF) >5% and a pre-determined linkage disequilibrium (LD) threshold of ≥0.8 in GIH population of HapMap. All tag-SNPs were genotyped using KASPar assays, which are competitive allele-polymerase chain reaction SNP genotyping assays using fluorescence resonance energy transfer (FRET) quencher cassette primers (KBioscience, Hoddesdon, UK). Amplifications were performed in Applied Biosystems PCR instrument (ABI Prism 9700, Foster City, CA, USA) and the fluorescent endpoints were measured using the ABI7900 SDS software (ABI Prism 7900, Foster City, CA, USA). The genotyping success rate was more than 99.5%.

### Ethical issues

After obtaining the ethics approval from the Institutional Ethics Committee, Sri Ramachandra University, Chennai, India, this research was conducted according to the principles* *of Declaration of Helsinki. Informed written consent was collected from all subjects before participating in the study.

### Statistical analysis

Genotype and allele frequencies were calculated, Hardy-Weinberg equilibrium was tested for the genotypes at each SNP by means of a chi-square test with one degree of freedom. Cochran*-*Armitage trend test was used to determine the association between ADPKD and *EDN1* tag-SNPs. Haplotypes and Pairwise LD were analysed using Haploview software version 4.1 (20). To assess the effect of *EDN1* genotypes and hypertension on CKD advancement, multivariate logistic regression analysis was performed within the ADPKD subjects. Mantel*-*Haenszel stratified analysis was performed by stratifying the study subjects based on the genotype and the relationship between different CKD stages and hypertension was assessed in each genotype. All the statistical analysis was were carried out using SPSS statistical software.

## Results

**Figure 1:**
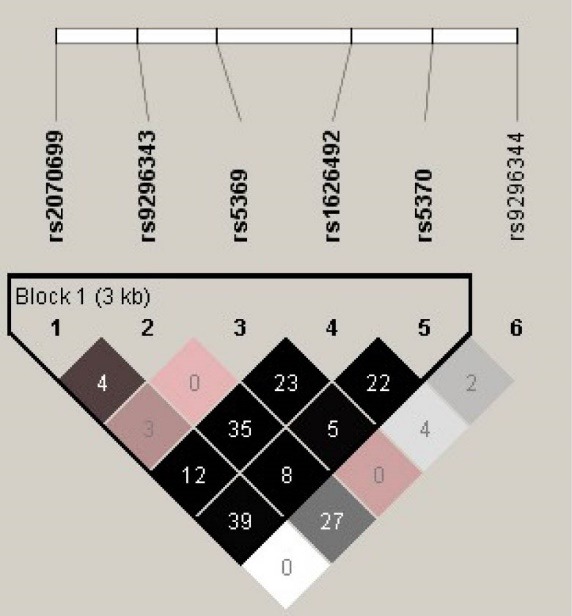
Pairwise LD measures (D′ and r^2^) between the markers. Square background represents the D′/LOD values, and the values in cells are r^2^ (multiplied by 100).

A total of 108 patients with ADPKD and 119 control subjects were included in the study. The mean age of control group was 53.1±12.5 years and ADPKD group was 46.9±11.4 years. For *EDN1* gene GIH population yielded six tag-SNPs (Rs2070699, rs9296343, rs5369, rs1626492, rs5370 and rs9296344). The allele and genotype frequencies of the six tag-SNPs were shown in Table 1. All polymorphisms followed Hardy-Weinberg equilibrium in both cases and controls. Distribution of *EDN1* genotypes between control and ADPKD groups was not statistically significant (Table 1). Analysis of LD revealed one haplotype block composed of 5 SNPs encompassing 3.4 kb, the r^2^ values indicating that the LD is strong between the markers pairs (Figure 1). The rs9296344 SNP located in 3’UTR remained outside the LD blocks. The haplotype distribution between ADPKD and controls was not statistically signiﬁcant (data not shown). Among ADPKD, 52 (48%) subjects showed advanced CKD stage with mean age of 51.0±9.8 years and 56 (52%) showed moderate progression with 43.0±11.6 years of age. Distribution of genotypes between preliminary and advanced CKD groups is not significant (Table 2). The effect of hypertension on CKD progression among different genotypes of the *EDN1* polymorphisms is almost similar and no confounding effect was observed (Table 3).

**Table 1:** Association between *EDN1* gene tag-SNPs and ADPKD patients

**SNP**	**Genotype**	**Control (%)** **(n=119)**	**ADPKD (%)** **(n=108)**	**OR (95% CI)**	***P*** **value** ^a^
Rs2070699	GG	61 (51.26)	44 (40.74)	1	
	TG	45 (37.82)	52 (48.15)	1.602 (0.918-2.794)	
	TT	13 (10.92)	12 (11.11)	1.280 (0.553-3.070)	0.229
HWp		0.291	0.563		
Rs9296343	CC	102 (85.71)	90 (83.33)	1	
	GC	15 (12.61)	16 (14.81)	1.209 (0.566-2.583)	
	GG	2 (1.68)	2 (1.85)	1.133 (0.553-8.211)	0.649
HWp		0.121	0.219		
Rs5369	GG	106 (89.08)	95 (87.96)	1	
	GA	12 (10.08)	13 (12.04)	1.209 (0.526-2.778)	
	AA	1 (0.84)	0 (0.0)	-	0.916
HWp		0.330	0.506		
Rs1626492	GG	75 (63.03)	67 (62.04)	1	
	GA	38 (31.93)	37 (34.26)	1.090 (0.623-1.908)	
	AA	6 (5.04)	4 (3.70)	0.746 (0.202-2.758)	0.964
HWp		0.680	0.688		
Rs5370	GG	36 (30.25)	37 (34.26)	1	
	TA	52 (43.70)	47 (43.52)	0.879 (0.480-1.611)	
	TT	31 (26.05)	24 (22.2)	0.753 (0.373-1.522)	0.430
HWp		0.174	0.225		
Rs9296344	TT	94 (78.99)	83 (76.85)	1	
	TC	23 (19.33)	23 (21.3)	1.133 (0.592-2.167)	
	CC	2 (1.68)	2 (1.85)	1.133 (0.516-8.220)	0.709
HWp		0.669	0.783		

Abbreviations: *EDN1*, endothelin 1; ADPKD, autosomal dominant polycystic kidney disease; SNP, single nucleotide polymorphism; HWp: Hardy-Weinberg equilibrium *P* value; OR, odds ratio. ^a^ *P* values for the Cochran-Armitage trend test.

**Table 2:** *EDN1* polymorphisms in ADPKD cases and their association with CKD stage

**Gene**	**Genotype**	**CKD stages**		***P*** **value** ^a^	**Adjusted** **OR (95% CI)**	***P*** **value** ^b^
		**Preliminary stage** ** No. (%)**	**Advance stage** **No. (%)**			
Rs2070699	GG	20 (35.7)	24 (46.2)		1	
	TG	29 (51.8)	23 (44.2)	0.292	0.606 (0.222, 1.657)	0.329
	TT	7 (12.5)	5 (9.6)		0.432 (0.084, 2.209)	0.313
rs9296343	CC	51 (91.1)	39 (75.0)		1	
	GC	4 (7.1)	12 (23.1)	0.052	2.487 (0.618, 10.0)	0.200
	GG	1 (1.8)	1 (1.9)		7.288 (0.255, 208.5)	0.246
rs5369	GG	48 (85.71)	47 (90.38)		1	
	GA	8 (14.29)	5 (9.62)	0.456	0.352 (0.076,1.625)	0.181
	AA	0 (0)	0 (0)		-	-
rs1626492	GG	39 (69.6)	28 (53.9)		1	
	GA	14 (25.0)	23 (44.2)	0.254	1.786 (0.632, 5.047)	0.274
	AA	3 (5.4)	1 (1.9)		0.730 (0.050,10.76)	0.819
rs5370	GG	20 (35.71)	17 (32.69)		1	
	TG	24 (42.86)	23 (44.23)	0.744	0.958 (0.334, 2.750)	0.937
	TT	12 (21.43)	12 (23.08)		1.606 (0.435, 5.932)	0.478
rs9296344	TT	46 (82.14)	37 (71.15)		1	
	TC	10 (17.86)	13 (25.0)	0.104	1.242 (0.401, 3.841)	0.707
	CC	0 (0.0)	2 (3.85)		-	-

Abbreviations: *EDN1*, endothelin 1; ADPKD, autosomal dominant polycystic kidney disease; CKD, chronic kidney disease; OR, odds ratio. ^a^ *P* values for the Cochran-Armitage trend test; ^b^Wald test *P* value.

**Table 3:** Association between CKD stages and hypertension stratified by *EDN1* genotypes

**Gene**	**Genotype**	**OR (95% CI) for HT**	***P*** **value** ^a^
rs2070699	GG	3.66 (0.63, 21.45)	0.837
	TG	4.73 (0.91, 24.60)	
	TT	-	
M-H combined	4.23 (1.27, 14.09)		
rs9296343	CC	2.69 (0.795, 9.121)	0.101
	GC	-	
	GG	-	
M-H combined	3.558 (1.13, 11.20)		
rs5369	GG	2.83 (0.82, 9.77)	0.235
	GA	-	
	M-H combined	3.79 (1.17, 12.29)		
rs1626492	GG	1.82 (0.428,7.77)	0.055
	GA	22.0 (2.29, 211.11)	
	AA	-	
M-H combined	4.18 (1.33, 13.10)		
rs5370	GG	-	0.076
	TG	0.952 (0.172, 5.28)	
	TT	0.50 (0.513, 59.01)	
M-H combined	3.78 (1.19, 12.05)		
rs9296344	TT	3.25 (0.96, 11.01)	0.516
	TC	-	
	CC	-	
M-H combined	3.61 (1.084, 11.99)		

Abbreviations: *EDN1*, endothelin 1; CKD, chronic kidney disease; HT, Hypertension; M-H, Mantel-Haenszel; OR, odds ratio. ^a^Homogeneity test *P* value.

## Discussion

Analysis of tag-SNPs within the *EDN1* gene in 108 ADPKD and 119 control subjects did not show significant association with ADPKD. LD was strong among all SNPs studied, covering a region of about 3.4 kb within the *EDN1* gene. Comparison of haplotypes between ADPKD and control groups also revealed the absence of a significant association with ADPKD. The *EDN1* genotypes are not contributing to the CKD advancement among the ADPKD patients.

It is well established that the processes that cause progression of CKD may be independent of the original insult. However the factors that induce the CKD progression in ADPKD include systemic and renal hypertension (21,22) with associated hyper-filtration (23), glomerular and tubular hypertrophy (24), and a combination of these leading to glomerulosclerosis and cortical tubulointerstitial fibrosis (25). Endothelin may contribute to all of these processes to modify the risk of developing CKD in ADPKD. In support of this hypothesis, elevated systemic and/or renal levels of endothelin have been noted clinically (26,27) and experimentally (28,29). Presence of immunoreactive ET1 in cyst epithelia, mesangial cells and vascular smooth muscle cells and neo-expression of 5 to 10-fold higher ETA mRNA in glomeruli and cysts suggesting continuing synthesis and action of ET1 in the cystic kidney (30). Endothelin exerts multiple and antagonistic effects on different aspects of renal physiology through its receptor subtypes, ETA and ETB. The balance between ETA and ETB signaling is important for maintaining tubular structure and function and act as a major modifying factor for cystic disease progression in human ADPKD (31,32). ET1 receptor antagonists have also been used to prevent the progression of CKD (33). Significant differences were observed in the plasma ET1 levels between ADPKD patients and control groups, while no significant differences were observed in ADPKD patients with or without hypertension (34).

The most compelling evidence of association was from rs9296343 and rs1626492 (IVS4) in the *EDN1* gene. Among them the rs9296343 is in tight LD with other SNP rs1800542 located within 50 base pair (bp) of splice acceptor sites in *EDN1* and potentially impact consensus binding sites for the exon splicing enhancers (35). The rs1626492 function is not clearly known but the carriers of this SNP suspected to be disadvantageous for survival of the original bleed following aneurysm rupture (36). Analysis of *EDN1* Lys198Asn and T1370G polymorphisms in nondiabetic subjects from the Netherlands revealed that the individuals with homozygous G-N haplotype (compound *EDN1*-1370GG/198NN genotype) have a lower glomerular filtration rate (GFR) than the remaining subjects (37). Analysis of K198N, 3A/4A, and T-1370G polymorphisms of *EDN1* in different groups of Czech ADPKD patients with ESRD did not show significant differences in their age among genotypes. But the haplotypes carrying 4A and 198N alleles showed significantly lower age at the time of ESRD (38).

Previous studies suggested additional mechanisms that may associated with renal disease progression. For example heterogeneous association of genetic variants with CKD in individuals with different lipid profiles has been postulated. In this regard, high serum HDL-cholesterol protects the individuals from developing PKD (39). More recently, epigenetic modifications have been proposed to play a role in both the susceptibility and progression to CKD.

## Conclusion

Our findings suggest that the *EDN1* gene tag-SNPs were not a major modifier of CKD advancement in ADPKD. However, endothelin-1 and endothelin receptor gene polymorphisms and their interactions with other genes and environmental factors should be analyzed in future investigations.

## Limitations of the study

One limitation of the present study is that the *GFR* estimation using creatinine-based mathematical equations. As serum creatinine concentration may not reflect the actual degree of kidney function of a particular subject, our results should be interpreted in the context of the clinical setting.

## Acknowledgements

The authors would like to thank Sri Ramachandra University for providing necessary facilities and the subjects.

## Authors’ contribution

BLVKS, RE and PS defined the research theme and designed methods. GR helped in sample collection. SNRA, BLVKS and RE performed genotyping, analyzed the data, interpreted the results and wrote the paper. All authors read and approved the final manuscript.

## Conflicts of interest

The authors declared no competing interests.

## Ethical considerations

Ethical issues (including plagiarism, data fabrication, double publication) have been completely observed by the authors.

## Funding/Support

No funding from any source.
